# Unveiling the phytochemical profiles of *Ziziphus jujuba* honey: An authenticity assurance approach

**DOI:** 10.1016/j.fochx.2025.102622

**Published:** 2025-06-03

**Authors:** Hequan Zhu, Liqiang Liu, Rongshen Wang, Zijing Wang, Yuesen Wang, Jie Dong, Qiqi Wang, Jiangtao Qiao, Hongcheng Zhang

**Affiliations:** a School of Life Sciences and Food Engineering, Hebei University of Engineering, Handan 056038, China; b State Key Laboratory of Resource Insects, Institute of Apicultural Research, Chinese Academy of Agricultural Sciences, Beijing 100093, China; c Shijiazhuang Center for Animal Disease Prevention and Control, Shijiazhuang, 130100, China; d College of Bee Science and Biomedicine, Fujian Agriculture and Forestry University, Fuzhou, 350002, China; e Hebei Ruiyuan Beekeeping Co., Ltd., Shijiazhuang 051230, China; f Key Laboratory of Bee Products for Quality and Safety Control, Ministry of Agriculture and Rural Affairs, Beijing 100093, China

**Keywords:** Jujube honey, Honey authenticity, Characteristic markers, HPLC fingerprints, Evaluation criteria

## Abstract

*Ziziphus jujuba* (jujube) honey, as a significantly agricultural product, faces persistent challenges in quality control due to the absence of reliable authentication techniques against adulteration. The research systematically identified and quantified phytochemicals using High-Performance Liquid Chromatography (HPLC) and High-Performance Liquid Chromatography-mass spectrometry (HPLC-MS). We identified and quantified 13 phytochemicals in the jujube honey samples, including 5 terpenoids, 4 phenolamides, 3 quinolines and 1 indole. Notably, four novel chemical markers were identified: 4-hydroxy-8-methoxyquinoline, vomifoliol, indole-3-carboxaldehyde, and jujubin ((E)-4-(2,3-dihydroxybutylidene)-3,5,5-trimethylcyclohex-2-en-1-one) can be regarded as the characteristic markers of jujube honey, with the average content of 10.25, 1.28, 1.87, and 0.58 mg/kg, respectively. To our knowledge, 4-hydroxy-8-methoxyquinoline, indole-3-carboxaldehyde, and jujubin are newly identified in honey products. Furthermore, the developed authentication methodology integrates quantitative analysis of these characteristic markers with HPLC fingerprint, successfully detecting adulteration in commercial samples. This approach appears to offer substantial improvements over conventional detection methods for the authentication of jujube honey.

## Highlights

• Three novel phytochemicals were identified for the first time in *Ziziphus jujuba* honey.
• Four specific markers were revealed in jujube honey.
• The authenticity of the commercial jujube honey was assessed by evaluation criteria.

## 1 Introduction

Honey is a natural sweetener produced by honeybees from the nectar of flowers or the secretions and excretions on the living parts of plants by plant-sucking insects. Honeybees collect these substances, blend them with their specific compounds, store the mixture, and allow it to ripen and mature in honeycombs (Abeshu & Geleta, 2016). Honey primarily contains approximately 60–78 % carbohydrates, 16–18 % water, and small amounts of protein, amino acids, vitamins, and other phytochemicals (Sindi et al., 2019). Since the Stone Age, this natural sweetener, characterized by its high energy content, has been part of the human diet for thousands of years. Anthropological research indicated that honey provided energy for ancient hominids and played a key role in their brain development (Crittenden, 2011). Honey has also been widely applied in the food industry as a raw material, such as beverages, baked goods, and confectionery. Nowadays, people increasingly prefer to consume honey directly due to its excellent biological activities, such as antioxidation, balancing gut microbiota, and anti-inflammation (Poulsen-Silva et al., 2023).

The global demand for honey has escalated in recent years due to an increased awareness of its health benefits and the growing consumer preference for natural products. However, the increasing demand for honey and its high price result in a lucrative market susceptible to adulteration (Craciun, Pârvulescu, Donise, Dobre, & Stanciu, 2020). Generally, honey adulteration occurs in two ways: by adding plant syrups and by mislabeling the plant or geographical origin (Moore, Brooks, Pappalardo, & Boufridi, 2025). Certain cheap syrups with a sugar composition similar to honey are directly added to it, such as corn sugar, high fructose corn and cane sugar syrup (Masoomi, Sharifi, & Hemmateenejad, 2024). Likewise, some low-priced, high-yield monofloral honeys are often mislabeled as premium varieties, and honey from developing countries is sometimes mislabeled as being from developed countries (Wang & Li, 2011). Furthermore, the most widely accepted international standards, such as the Codex Alimentarius Standard and the European Honey Council Directive 2001/110/EC (Council, 2002), only allow mixtures of pure honey. Noteworthily, Apimondia specified three other possible ways in which honey can be adulterated (Apimondia, 2019). These possible ways include harvesting and dehydrating immature honey, removing residues such as 5-hydroxymethylfurfural (HMF) by ion-exchange resins, and artificial feeding of bees during a nectar flow. This fraudulent behavior destroys the honey market's integrity, damages the beekeepers' enthusiasm, and harms the ecosystem. Therefore, identifying the authenticity of honey is beneficial to the honey market and ecological balance.

Uncovering honey adulteration is a formidable challenge due to the natural variability in the composition of genuine honey. Traditional authentication solutions, such as physics, chemistry and melissopalynology analysis, have proven inadequate in addressing the sophistication of modern adulteration techniques (Massaro et al., 2024; Qiao et al., 2020). The official ^13^C/^12^C- isotope ratio mass spectrometry (IRMS) reliably detects adulteration with C4 plant syrups, for example, cane and corn syrups, but fails to identify C3 plant syrups, like wheat, rice and beet syrups, due to similar isotope ratios to honey (Wu et al., 2017). Therefore, the current official Association of Official Analytical Chemists (AOAC) 998.12 standard sometimes white-wash adulterated honey (Zábrodská & Vorlová, 2014). Melissopalynology, the microscopic pollen analysis of honey, is a relatively reliable approach to identifying honey's botanical and geographical origins by providing direct evidence of the botanical sources from which bees collect nectar. However, this technique has limitations, as its results can be influenced by plant morphology, bee foraging behavior, and contamination during hive management or processing. Additionally, researchers have also developed several instrumental methods to assess honey authenticity, such as mass spectrometry (MS) analysis (Manickavasagam, Saaid, & Lim, 2024), nuclear magnetic resonance (NMR) spectroscopy (Elisabetta Schievano, Sbrizza, Zuccato, Piana, & Tessari, 2020), and optical spectroscopy (Oroian, Ropciuc, & Paduret, 2018). MS analysis generally includes liquid chromatography-mass spectrometry (LC-MS) and gas chromatography–mass spectrometry (GC–MS), which can identify aldehydes, esters, terpenes, phenolic acids and flavonoids in honey (Manickavasagam et al., 2024). NMR can effectively determine the sugar composition of honey and provide carbohydrate profiles of honey (Leoni et al., 2024; Elisabetta Schievano et al., 2020; Schievano, Tonoli, & Rastrelli, 2017). Another method for identifying syrup adulterants in honey is optical spectroscopy, involving Raman and Fourier transform infrared spectroscopy (Oroian et al., 2018; Se, Ghoshal, Wahab, Ibrahim, & Lani, 2018). However, all three methods rely heavily on large databases, complex algorithms, skilled analysts, and specialized equipment. Recently, researchers proposed a more straightforward and reliable method to assess honey authenticity based on the qualitative and quantitative analysis of its intrinsic characteristic phytochemicals (Vazquez, Armada, Celeiro, Dagnac, & Llompart, 2021).

Phytochemicals are secondary metabolites that enable plants to interact with the biotic and abiotic environments. Phytochemicals are widely found in plants' roots, stems, leaves, flowers, and nectaries (Godlewska et al., 2022). These compounds are transferred from nectaries into honey through the collecting action of the honeybees (Hájek, 2023). Each plant possesses a unique metabolic profile, resulting in significant variation in the types and amounts of phytochemicals across different monofloral honeys. Therefore, some intrinsic phytochemicals are considered specific markers to distinguish honey plant origin. These phytochemicals in honey mainly include phenolic acid, flavonoids and terpenes (Adedayo et al., 2021). Recently, several distinctive phytochemicals have been identified as markers of monofloral honeys; for instance, phaseic acid in acacia honey (Qiao et al., 2020), kaempferitrin in *camellia oleifera* honey (Z. Li et al., 2023), and unedone in bauhinia honey (Du et al., 2023). Jujube honey, derived from *Ziziphus* plants, is widely distributed worldwide (Bouddine et al., 2024; Zerrouk, Seijo, Escuredo, & Rodríguez-Flores, 2018; Zhang et al., 2019). As one of the most valued and popular varieties, jujube honey is attributed to its desirable taste and flavor (Zhang et al., 2019). Chinese jujube honey, originating from *Ziziphus jujuba* Mill., is among the most produced and consumed types of jujube honey globally. The price of Chinese jujube honey is significantly higher than that of other monofloral honeys, leading to widespread adulteration.

Current authentication systems for *Ziziphus jujuba* (jujube) honey confront two critical limitations: (1) inherent technical constraints of conventional analytical methods in detecting advanced adulteration strategies, and (2) inadequate chemical characterization and validation of its unique phytochemical profile. To resolve these dual challenges, we implemented an advanced chromatographic workflow combining targeted and untargeted analytical modalities. Our methodological framework incorporated dual analytical approaches: High-Performance Liquid Chromatography-Quadrupole Time-of-Flight Tandem Mass Spectrometry (HPLC-QTOF-MS/MS) for comprehensive compound identification, coupled with High-Performance Liquid Chromatography-Photo-Diode Array (HPLC-PDA) detection for precise quantitative analysis. Furthermore, we developed a standardized High-Performance Liquid Chromatography (HPLC) phytochemical fingerprint utilizing the ‘Similarity Evaluation System for Chromatographic Fingerprint of TCM (Traditional Chinese Medicine)’. The integration of these analytical strategies enabled the proposal of a novel authenticity assessment protocol, combining both targeted quantification of marker compounds and nontargeted chromatographic pattern recognition. This multi-dimensional approach significantly enhances the scientific foundation for jujube honey authentication while providing practical tools for quality control implementation.

## 2 Materials and methods

### 2.1 Reagents

Standard references of 4-hydroxyquinoline (S66874, ≥98.0 %), 4-hydroxy-8-methoxyquinoline (Y30968, ≥98.0 %), vomifoliol (B30361, ≥97.0 %), 2-hydroxyquinoline (B24635, ≥98.0 %), indole-3-carboxaldehyde (B27461, ≥98.0 %), phaseic acid (B38362, ≥98.0 %) were obtained from Shanghai Yuanye Biotechnology Co., Ltd. (Shanghai, China). *cis*, *trans*-Abscisic acid (862,169, ≥98.0 %) and *trans*, *trans*-abscisic acid (A110025, ≥98.0 %) were respectively purchased from Sigma-Aldrich Chemical Co., Ltd. (St. Louis, MO) and Toronto Research Chemicals. N1(Z), N5(Z), N10(*Z*)-tri-*p*-coumaroyl spermidine (purity≥97.0 %) and jujubin (purity≥97.0 %) were prepared using preparative HPLC by ourselves.

Chromatographic-grade methanol (Optima™ LC/MS, Fisher Chemical) and acetic acid (LC-MS certified) were acquired from Thermo Fisher Scientific (Fair Lawn, NJ), with methanol additionally sourced in analytical grade from Sinopharm Chemical Reagent Co., Ltd. (Shanghai, China). Solid-phase extraction was performed using Strata™-X-A mixed-mode Solid Phase Extraction (SPE) column cartridges (60 mg/3 mL, P/N 8B-S029-FBJ) from Phenomenex (Torrance, CA). Ultrapure water (18.2 MΩ·cm) was generated using a Milli-Q® Integral 3 system (Merck Millipore, Billerica, MA) equipped with a 0.22 μm Q-POD® Pak filter.

### 2.2 Honey samples

Fifteen samples of raw jujube honey were from *Apis mellifera* apiaries across 5 provinces in China in 2022. The plant origin of these samples was declared and recorded by professional beekeepers and our researchers based on the hive positions and floral sources of the bee forage area. Melissopalynology described by Lutier and Vaissière (Lutier & Vaissière, 1993) was validated using phase contrast microscopy. The sample information is listed in Table S1, and the pollen micrographs of these raw honeys are shown in Fig. S1.

Fifteen commercial jujube honey samples were randomly purchased from various brands. All samples were stored at −20 °C for further analysis.

### 2.3 Enrichments of honey phytochemicals

Identification of honey phytochemicals is challenging owing to their low concentrations at the parts per million (ppm) level. Thus, pre-concentrating phytochemicals is essential for improving the accuracy and reliability of HPLC analysis. Strata-X-A SPE cartridges were used for phytochemical preconcentration following our earlier method (Sun, Tan, Zhang, & Zhang, 2016; Zhu et al., 2024). First, the honey sample (20 g) was dissolved in ultrapure water (80 mL), and then the solution pH was adjusted to 6.5 using 5 % ammonium (v/v). Subsequently, the solution was centrifuged to remove pollen granules at 10,000 ×*g* for 20 min at 4 °C, and the supernatant was collected. At the same time, the Strata-X-A cartridges were successively activated with methanol (3 mL) and balanced with ultrapure water (3 mL). The supernatant is added to the Strata-X-A cartridge, and once it completely passes through, the phytochemicals are adsorbed onto the Strata-X-A cartridge. Subsequently, the cartridge was washed to remove unabsorbed substances with ultrapure water (3 mL), and then 3 mL of formic acid: methanol (1:9, v/v) was used to elute the phytochemicals from the cartridge. The eluate was gathered in a centrifuge tube and dried with a nitrogen blower (YDCY-24 L, Shanghai Xiyang Instrument Co., Ltd., Shanghai, China). Finally, the dried residue was redissolved in methanol (2 mL) with 2 % acetic acid and filtered through a 0.22 μm filter (MILLEX-GA, Millipore, Ireland) before HPLC analysis.

### 2.4 Phytochemicals detection via HPLC-PDA

HPLC-PDA was used for phytochemicals detection according to our previous method (Zhu et al., 2024), and the elution procedure was optimized to enhance resolution of various components in jujube honey. Chromatographic separation was performed on a Shimadzu HPLC system equipped with the following modules: PDA-20 A diode array detector (190–800 nm spectral range), SIL autosampler, CTO-10 AC column oven (± 0.1 °C temperature control), LC-20ADXR binary pump (0.001–10 mL/min flow range). Separation was achieved using a Gemini® C18 column (150 × 4.6 mm, 3 μm from Phenomenex. The mobile phase system consisted of: Phase A (Ultrapure water containing 2 % (*v*/v) acetic acid (LC-MS grade, ≥99.9 %)); Phase B (Methanol (HPLC gradient grade, ≥99.93 %) with 2 % (v/v) acetic acid modifier). A seven-segment gradient elution protocol was implemented as follows: 0–15 min, 3–5 % B; 15–25 min, 5–9 % B; 25–55 min, 9–18 % B; 55–85 min, 18–21 % B; 85–120 min, 21–45 % B; 120–140 min, 45–54 % B; 140–160 min, 54–80 % B. The separation was conducted at a flow rate of 0.75 mL/min with the column oven maintaining the temperature at 35.0 °C (± 0.1 °C stability). Post-run equilibration employed 5-column volumes of initial conditions. The quantitative analysis utilized multi-point external calibration curves, at a wavelength of 260 nm (R^2^ > 0.999).

### 2.5 High-resolution phytochemicals via HPLC-QTOF-MS/MS

HPLC-QTOF-MS/MS was used for high-resolution phytochemicals analysis according to our previous method (Qiao et al., 2020). Compound characterization was conducted using an Agilent 6520 Accurate-Mass Q-TOF LC/MS system (Santa Clara, CA) configured with Dual AJS electrospray ionization (ESI) source and MassHunter Workstation (v.B.08.00). Chromatographic separation followed the gradient elution protocol detailed in Section 2.4. Mass spectrometric detection parameters were optimized as follows: Ionization: Dual ESI (±) mode with sheath gas (11 L/min, 350 °C) and drying gas (7 L/min, 325 °C). Voltage settings: Nozzle 500 V, capillary 130 V, fragmentor 380 V. MS operation: Full scan (*m*/*z* 50–900) at 2 GHz extended dynamic range. MS/MS acquisition: Targeted mode with fixed collision energies (20 eV) and dynamic auto CID adjustment. A universal dual-nebulizer ESI source was employed with reference ions (m/z 121.0509 and 922.0098) to achieve reference mass correction (Conceição Meira Silva et al., 2024).

### 2.6 Semipreparative isolation of reference standards

The target compounds N1(Z), N5(Z), N10(*Z*)-tri-*p*-coumaroyl spermidine and jujubin were prepared through phytochemical enrichment followed by chromatographic separation. Isolation was performed on an LC-6 CE semipreparative HPLC system (Shimadzu) integrated with an SPD-M20A photodiode array detector and FRC-10 A fraction collector. Chromatographic separation was achieved using a Shim-pack Prep-ODS (H) column (250 × 20 mm, 5 μm; Cat. No. 228–41,755-20) maintained at 35 °C with the following phase system: Mobile Phase A: Ultrapure water (18.2 MΩ·cm) acidified with 2 % (*v*/v) formic acid (LC-MS grade). Mobile Phase B: Methanol (Optima™ LC/MS) containing 2 % (v/v) formic acid.

The chromatographic separation was performed under isocratic conditions with a mobile phase flow rate of 3.0 mL/min. Before injection, synthesized crude extracts were filtered through hydrophilic polytetrafluoroethylene (PTFE) membranes (0.22 μm, Merck Millipore, Cat. No. SLGV033RB) using American Society of Testing Materials (ASTM) D4196–82 standard filtration procedures. Fraction collection was triggered by ultraviolet (UV) absorption at 260 nm with 5 mL injection volumes per run.

### 2.7 Statistics

Triplicate phytochemical quantification trials were conducted to ensure analytical precision. Numerical datasets underwent statistical processing through IBM's SPSS Statistics Suite (Release 21.0, Armonk, NY), employing parametric analysis models. Quantitative results were expressed using descriptive statistics (arithmetic mean ± population standard deviation). Chromatographic similarity and HPLC fingerprint analysis were performed utilizing the “Similarity Evaluation System for TCM Chromatographic Fingerprints (Version 2012 A).

## 3 Results

### 3.1 Identification of characteristic components

**Fig. 1:**
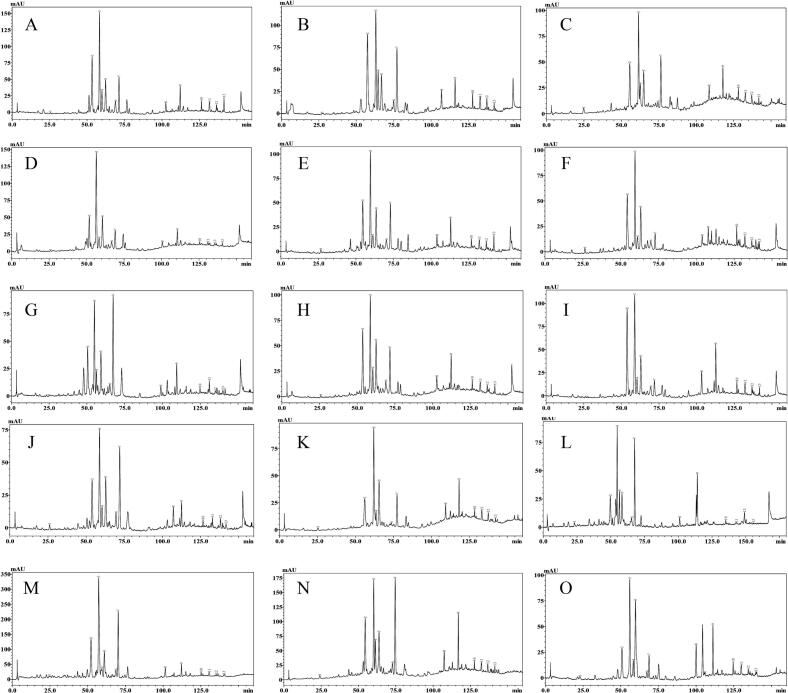
HPLC profiles of raw jujube honey samples (260 nm). Note: 1: 4-Hydroxyquinoline; 2: 4-Hydroxy-8-methoxyquinoline; 3: Vomifoliol; 4: 2-Hydroxyquinoline; 5: Indole-3-carboxaldehyde; 6: Jujubin; 7: Phaseic acid; 8: *Trans, trans*-abscisic acid;9: *Cis, trans*-abscisic acid; 10: N1(Z), N5(Z), N10(*Z*)-tri-*p*-coumaroyl spermidine; 11: N1(Z), N5(Z), N10(*E*)-tri-*p*-coumaroyl spermidine; 12: N1(E), N5(Z), N10(E)-tri-*p*-coumaroyl spermidine; and 13: N1(E), N5(E), N10(E)-tri-*p*-coumaroyl spermidine.

**Fig. 2:**
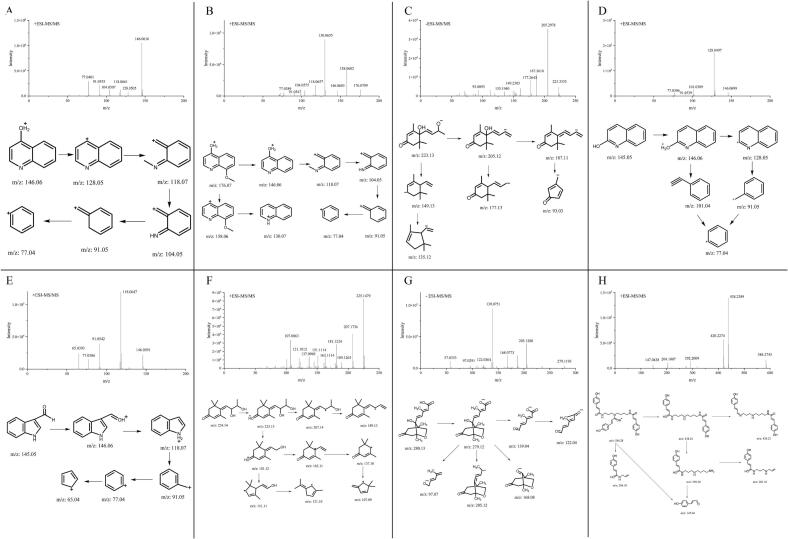
MS/MS spectra and fragment cleavage patterns of eight compounds. Note: A: 4-Hydroxyquinoline; B: 4-Hydroxy-8-methoxyquinoline; C: Vomifoliol; D: 2-Hydroxyquinoline; E: Indole-3-carboxaldehyde; F: Jujubin; G: Phaseic acid; and H: tri-*p*-Coumaroyl spermidine and its isomers.

The HPLC profiles of raw jujube honey samples are shown in Fig. 1. For the phytochemicals in the honey samples, the cleavage patterns and ion fragmentations of their mass spectra are presented in Fig. 2 and Table 1.

**Table 1:** Phytochemicals identified in honeys and their UV and MS characteristics.

Peak	Compounds identified	Formula	UV λ_max_(nm)	Mw	Ion fragments (*m*/*z*)
1	4-Hydroxyquinoline	C_9_H_7_NO	310	145	146, 128, 118, 104,91,77 (MS/MS^+^)
2	4-Hydroxy-8-methoxyquinoline	C_10_H_9_NO_2_	260,302	175	176, 158, 146, 130, 118, 104, 91, 77 (MS/MS^+^)
3	Vomifoliol	C_13_H_20_O_3_	237	224	223, 205, 187, 177, 149, 135, 93 (MS/MS^−^)
4	2-Hydroxyquinoline	C_9_H_7_NO	313	145	146, 128, 101, 91,77 (MS/MS^+^)
5	Indole-3-carboxaldehyde	C_9_H_7_NO	262,300	145	146, 118, 91, 77, 65 (MS/MS^+^)
6	Jujubin	C_13_H_20_O_3_	287	224	225, 207, 189, 181, 163, 151, 137, 121, 107 (MS/MS^+^)
7	Phaseic acid	C_15_H_20_O_5_	264	280	279, 205, 168, 139, 122, 97 (MS/MS^−^)
8	*Trans, trans*-abscisic acid	C_15_H_20_O_4_	265	264	265, 187, 168, 157, 68 (MS/MS^+^)
9	*Cis, trans*-abscisic acid	C15H20O4	265	264	265, 187, 168, 157, 68 (MS/MS^+^)
10	N1(Z), N5(Z), N10(Z)-tri-*p*-coumaroyl spermidine	C_34_H_37_N_3_O_6_	269	583	584, 438, 292, 275, 204, 218, 147 (MS/MS^+^)
11	N1(Z), N5(Z), N10(E)-tri-*p*-coumaroyl spermidine	C_34_H_37_N_3_O_6_	278	583	584, 438, 292, 275, 204, 218, 147 (MS/MS^+^)
12	N1(E), N5(Z), N10(E)-tri-*p*-coumaroyl spermidine	C_34_H_37_N_3_O_6_	290	583	584, 438, 292, 275, 204, 218, 147 (MS/MS^+^)
13	N1(E), N5(E), N10(E)-tri-*p*-coumaroyl spermidine	C_34_H_37_N_3_O_6_	297	583	584, 438, 292, 275, 204, 218, 147 (MS/MS^+^)

#### 3.1.1 4-Hydroxyquinoline and 2-Hydroxyquinoline

Compounds 1 and 4 shared similar +ESI-MSMS spectra and cleavage patterns demonstrated in Fig. 2A and D. The quasi-molecular ion at *m*/*z* 146 [M + H]^+^ can speculate the chemical formula of compounds 1 and 4 as C_9_H_7_NO, and the degree of unsaturation was calculated as 7, indicating that they are ring compounds. The quasi-molecular ion at *m*/*z* 146 was dehydrated via the loss of 18 amu to generate fragment ions at m/z 128 [M + H − H_2_O]^+^. Since the unsaturation degree of compound 1 is 7, it can be inferred that it contains a quinoline structure. Therefore, the compound 1 and 4 were identified as hydroxyquinoline. In Fig. 2A, fragment ions at *m*/*z* 128 [M + H − H_2_O]^+^ underwent electronic rearrangement, yielding fragment ions at m/z 118 (C_8_H_8_N^+^). Then the fragment ions at m/z 118 (C_8_H_8_N^+^) successively broken into ion fragments at m/z 104 (C_7_H_6_N^+^), 91 (C_7_H_7_^+^) and 77 (C_6_H_5_^+^). In Fig. 2D, the quasi-molecular ion at *m*/*z* 146 [M + H]^+^ underwent electronic rearrangement and directly generated the ion fragments at *m*/*z* 101 (C_8_H_5_^+^) due to the instability of the enol structure. Moreover, the maximum UV absorption wavelengths of compounds 1 and 4 varied. The maximum UV absorption wavelength of compound 1 was 310 nm, while compound 4 was 313 nm. Finally, compounds 1 and 4 were identified as 4-hydroxyquinoline and 2-hydroxyquinoline according to the database (Consortium & Contributors, 2024) and verified by commercial standards.

#### 3.1.2 4-Hydroxy-8-methoxyquinoline

The +ESI-MSMS spectrum of compound 2 is demonstrated in Fig. 2B. Compound 2 chemical formula was determined as C_10_H_9_NO_2_ by the quasi-molecular ion at *m*/*z* 176 [M + H]^+^ in Fig. 2B. The quasi-molecular ion at m/z 176 generated fragment ions at *m*/*z* 146 [M + H − OCH_3_]^+^, losing 30 amu (−methoxy group). The subsequent fragmentation pattern closely resembled that of compound 1, indicating that compound 2 contains an additional methoxy group based on the quinoline structure of compound 1. Moreover, the quasi-molecular ion at *m*/*z* 176 lost hydroxyl and methoxy groups, yielding the fragment ions at m/z 158 [C_10_H_8_NO^+^] and 130 [C_9_H_8_N^+^]. The maximum UV absorption wavelength of compound 2 were 260 and 302 nm. Compound 2 was validated as a 4-hydroxy-8-methoxyquinoline by commercial standards.

#### 3.1.3 Vomifoliol

The -ESI-MSMS spectrum of compound 3 is demonstrated in Fig. 2C. The compound 3 chemical formula was determined as C_13_H_20_O_3_ by the quasi-molecular ion at *m*/*z* 223 [M - H]^−^. The quasi-molecular ion at *m*/*z* 223 lost one and two H_2_O residues and respectively produced fragment ions at *m*/*z* 205 (C_13_H_17_O_2_^−^) and m/z 187 (C_13_H_15_O^−^), indicating that compound 3 had two hydroxyl groups. These two ion fragments underwent electronic rearrangement, removing the CO residue and C_7_H_10_ residue to produce ion fragments at *m*/*z* 177 (C_12_H_17_O^−^) and m/z 93 (C_6_H_5_O^−^), respectively. The ion fragments at m/z 149 (C_11_H_17_^−^) were also generated from quasi-molecular ions at m/z 223 and subsequently generated the ion fragment at m/z 135 (C_10_H_15_^−^). Compound 3 displayed maximum UV absorption wavelengths at 237 nm and was validated as a vomifoliol by commercial standards.

#### 3.1.4 Indole-3-carboxaldehyde

The +ESI-MSMS spectrum of compound 5 is demonstrated in Fig. 2E. The quasi-molecular ion at *m*/*z* 146 [M + H]^+^ can speculate the chemical formula of compound 5 as C_9_H_7_NO. The quasi-molecular ion at *m*/*z* 146 lost 28 amu (−carbonyl group) to generate fragment ions at *m*/*z* 118 [M + H − CO]^+^. The fragment ion at *m*/*z* 118 underwent McLaren rearrangement fragmentation, losing a CHN ion to yield a fragment ion at *m*/*z* 91(C_7_H_7_^+^), and then this fragment ion lost C_2_H_2_ to obtain m/z 65 (C_5_H_5_^+^). Compound 5 displayed maximum UV absorption wavelengths at 262 and 300 nm and was validated as indole-3-carboxaldehyde by commercial standards.

#### 3.1.5 Jujubin

The +ESI-MSMS spectrum of compound 6 is demonstrated in Fig. 2F. Compound 6 chemical formula was determined as C_13_H_20_O_3_ by the quasi-molecular ion at *m*/*z* 225 [M + H]^+^. The quasi-molecular ion at *m*/*z* 225 lost a CH_3_CHO residue to produce a fragment ion at m/z 181 (C_11_H_17_O_2_^+^), followed by the successive loss of two CH_3_OH residues to produce fragment ions at *m*/*z* 151 (C_10_H_15_O^+^) and *m*/*z* 121(C_9_H_13_^+^), respectively. The fragment ion at m/z 181 (C_11_H_17_O_2_^+^) lost one H_2_O residue and two CH_3_ residues, generating fragment ions at *m*/*z* 163 (C_11_H_15_O^+^) and m/z 137 (C_9_H_13_O^+^), respectively. The fragment ions at m/z 137 (C_9_H_13_O^+^) then loss one CH_3_OH residue to generate the fragment ions at m/z 107 (C_8_H_11_^+^). This compound displayed maximum UV absorption wavelengths at 287 nm. Furthermore, the compound C_13_H_20_O_3_ was identified as (E)-4-(2,3-dihydroxybutylidene)-3,5,5-trimethylcyclohex-2-en-1-one. We named it jujubin because of its unique presence in jujube honey.

#### 3.1.6 Phaseic acid

The -ESI-MSMS spectrum of compound 7 is demonstrated in Fig. 2G. Compound 7 chemical formula was determined as C_15_H_20_O_5_ by the quasi-molecular ion at m/z 279 [M - H]^−^. In the negative ionization mode, the quasi-molecular ion at m/z 279 lost the C_8_H_12_O_2_ residue and the OH residue sequentially to produce ion fragments at m/z139 and m/z122.The quasi-molecular ion at m/z 279 also produced three other ion fragments at m/z 205 (C_13_H_17_O_2_^−^), m/z 168 (C_9_H_12_O_3_^−^), and m/z 97 (C_6_H_9_O^−^), respectively. This compound displayed maximum UV absorption wavelengths at 264 nm and was validated as phaseic acid by commercial standards.

#### 3.1.7 Tri-*p*-Coumaroyl spermidine

The +ESI-MSMS spectra and cleavage patterns of compounds 10–13 are displayed in Fig. 2H and exhibited the same fragmentation pattern with the same quasi-molecular ion [M + H]^+^ at m/z 584. The quasi-molecular ion at m/z 584 lost 146 amu (*p*-coumaroyl residue) to generate three ion fragments at m/z 438 (C_25_H_32_N_3_O_4_^+^). The fragment ion at m/z 438 then lost another 146 amu (*p*-coumaroyl residue) to generate the fragment ion at m/z 292 [M + H − C_9_H_6_O_2_ − C_9_H_6_O_2_]^+^, demonstrating the existence of two *p*-coumaroyl residue. The ion fragment at m/z 147 (C_9_H_7_O_2_^+^) also verified the existence of the *p*-coumaroyl residue. The fragment ion at m/z 204 fragments was the characteristic fragment ion of *p*-coumaroyl spermidine. Compounds 10–13 exhibited different maximum UV absorption wavelengths at 269, 278, 290 and 297 nm, respectively. The compound 10 was inferred as N1(Z), N5(Z), N10(*Z*)-tri-*p*-coumaroyl spermidine according to the previous report (Qiao et al., 2023) and validated by the prepared standard. The compound 11–13 were identified as N1(Z), N5(Z), N10(*E*)-tri-*p*-coumaroyl spermidine, N1(E), N5(Z), N10(E)-tri-*p*-coumaroyl spermidine and N1(E), N5(E), N10(E)-tri-*p*-coumaroyl spermidine based on retention time and maximum UV absorption wavelength (Du et al., 2023).

### 3.2 Phytochemical Profiles of Raw Jujube Honey Samples

HPLC profiles of jujube honey samples are demonstrated in Fig. 1, and 13 phytochemicals were identified in Table 1 and quantified in Table 2 by comparison with those of commercial and prepared standards. The highest chromatographic peak, labeled as peak 3, was identified as vomifoliol in Fig. 1. The average content of vomifoliol was 10.25 mg/kg, ranging from 4.50 to 18.25 mg/kg (Table 2). The chromatographic peaks 2, 5, and 6 were also remarkable and identified as 4-hydroxy-8-methoxyquinoline, indole-3-carboxaldehyde, and jujubin, respectively. The average contents of 4-hydroxy-8-methoxyquinoline, indole-3-carboxaldehyde, and jujubin were 1.28, 1.87, and 0.58 mg/kg, respectively (Table 2). Jujube honey can be characterized by the high concentrations of vomifoliol and the presence of 4-hydroxy-8-methoxyquinoline, indole-3-carboxaldehyde, and jujubin. Thus, these four compounds can be identified as the intrinsic markers of jujube honey.

**Table 2:** Contents of characteristic compounds in raw honey samples (mg/kg).

Compound	S1	S2	S3	S4	S5	S6	S7	S8
4-Hydroxyquinoline	0.25 ± 0.01	0.26 ± 0.01	0.40 ± 0.02	0.21 ± 0.01	0.18 ± 0.01	0.15 ± 0.01	0.22 ± 0.01	0.20 ± 0.01
4-Hydroxy-8-methoxyquinoline	1.42 ± 0.12	1.25 ± 0.09	1.32 ± 0.08	1.29 ± 0.08	1.08 ± 0.07	0.97 ± 0.04	1.16 ± 0.09	1.12 ± 0.07
Vomifoliol	11.08 ± 0.34	10.04 ± 0.85	7.29 ± 0.48	11.91 ± 0.87	9.47 ± 0.49	8.83 ± 0.47	7.57 ± 0.39	11.12 ± 0.71
2-Hydroxyquinoline	1.21 ± 0.11	1.45 ± 0.09	0.88 ± 0.06	1.09 ± 0.08	1.13 ± 0.08	1.15 ± 0.10	0.42 ± 0.02	1.13 ± 0.07
Indole-3-carboxaldehyde	1.66 ± 0.12	2.01 ± 0.16	1.73 ± 0.13	1.68 ± 0.13	1.70 ± 0.14	1.48 ± 0.08	1.74 ± 0.13	2.02 ± 0.16
Jujubin	0.97 ± 0.06	1.06 ± 0.06	0.26 ± 0.02	0.49 ± 0.02	0.88 ± 0.06	0.67 ± 0.04	0.32 ± 0.02	0.44 ± 0.02
Phaseic acid	1.16 ± 0.12	2.04 ± 0.17	1.16 ± 0.07	1.25 ± 0.11	1.27 ± 0.09	0.70 ± 0.06	2.50 ± 0.14	1.37 ± 0.10
*Trans, trans*-abscisic acid	0.24 ± 0.02	0.45 ± 0.04	0.39 ± 0.02	0.29 ± 0.02	0.18 ± 0.02	0.97 ± 0.06	0.11 ± 0.01	0.29 ± 0.01
*Cis, trans*-abscisic acid	0.74 ± 0.05	0.59 ± 0.03	0.49 ± 0.04	0.62 ± 0.03	0.46 ± 0.03	1.65 ± 0.12	0.69 ± 0.04	0.73 ± 0.06
N1(Z), N5(Z), N10(Z)-tri*-p*-coumaroyl spermidine	0.72 ± 0.04	0.60 ± 0.04	0.65 ± 0.05	0.35 ± 0.02	0.48 ± 0.02	0.74 ± 0.06	0.64 ± 0.04	0.45 ± 0.03
N1(Z), N5(Z), N10(E)-tri-*p*-coumaroyl spermidine	1.32 ± 0.11	0.64 ± 0.04	0.89 ± 0.06	0.56 ± 0.03	0.89 ± 0.06	0.88 ± 0.07	0.72 ± 0.05	0.89 ± 0.06
N1(E), N5(Z), N10(E)-tri-*p*-coumaroyl spermidine	0.90 ± 0.07	1.08 ± 0.08	0.88 ± 0.06	0.52 ± 0.04	0.78 ± 0.04	0.63 ± 0.04	0.58 ± 0.03	0.75 ± 0.05
N1(E), N5(E), N10(E)-tri-*p*-coumaroyl spermidine	1.51 ± 0.11	0.65 ± 0.05	0.60 ± 0.06	0.53 ± 0.03	1.08 ± 0.07	0.60 ± 0.03	0.56 ± 0.04	0.74 ± 0.05

Compound	S9	S10	S11	S12	S13	S14	S15	
4-Hydroxyquinoline	0.21 ± 0.01	0.18 ± 0.01	0.35 ± 0.02	0.13 ± 0.01	0.97 ± 0.07	0.33 ± 0.02	0.86 ± 0.08	
4-Hydroxy-8-methoxyquinoline	1.20 ± 0.09	0.98 ± 0.08	1.38 ± 0.11	1.32 ± 0.09	1.76 ± 0.12	0.65 ± 0.04	2.25 ± 0.19	
Vomifoliol	9.80 ± 0.62	12.33 ± 0.96	6.98 ± 0.56	4.50 ± 0.29	18.25 ± 1.15	12.58 ± 0.92	11.97 ± 0.83	
2-Hydroxyquinoline	0.89 ± 0.06	0.71 ± 0.04	0.52 ± 0.03	0.74 ± 0.05	1.48 ± 0.09	1.71 ± 0.13	0.81 ± 0.05	
Indole-3-carboxaldehyde	1.34 ± 0.08	2.16 ± 0.14	1.47 ± 0.13	1.82 ± 0.12	2.39 ± 0.16	2.48 ± 0.13	2.39 ± 0.17	
Jujubin	0.55 ± 0.02	0.65 ± 0.04	0.15 ± 0.01	1.27 ± 0.07	0.36 ± 0.02	0.46 ± 0.03	0.18 ± 0.01	
Phaseic acid	0.35 ± 0.02	1.94 ± 0.13	0.64 ± 0.04	1.60 ± 0.11	5.43 ± 0.32	4.18 ± 0.19	0.37 ± 0.02	
*Trans, trans*-abscisic acid	0.86 ± 0.06	0.34 ± 0.02	0.37 ± 0.02	0.12 ± 0.01	0.60 ± 0.03	0.84 ± 0.06	0.70 ± 0.04	
*Cis, trans*-abscisic acid	1.36 ± 0.09	0.51 ± 0.03	0.67 ± 0.02	0.98 ± 0.05	0.82 ± 0.04	1.65 ± 0.12	0.87 ± 0.05	
N1(Z), N5(Z), N10(Z)-tri-*p*-coumaroyl spermidine	0.77 ± 0.05	0.48 ± 0.03	0.56 ± 0.03	0.32 ± 0.02	0.95 ± 0.07	0.66 ± 0.04	0.53 ± 0.03	
N1(Z), N5(Z), N10(E)-tri-*p*-coumaroyl spermidine	1.24 ± 0.08	0.57 ± 0.03	0.77 ± 0.05	0.27 ± 0.02	0.87 ± 0.05	1.16 ± 0.09	0.85 ± 0.04	
N1(E), N5(Z), N10(E)-tri-*p*-coumaroyl spermidine	0.83 ± 0.05	0.51 ± 0.03	0.71 ± 0.06	0.30 ± 0.02	0.97 ± 0.05	1.16 ± 0.08	0.68 ± 0.04	
N1(E), N5(E), N10(E)-tri-*p*-coumaroyl spermidine	0.73 ± 0.04	0.47 ± 0.03	0.36 ± 0.02	0.22 ± 0.01	0.62 ± 0.04	1.00 ± 0.07	0.38 ± 0.02	

**Fig. 3:**
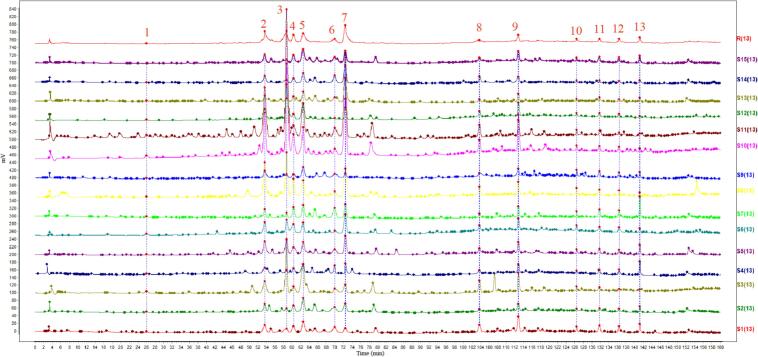
HPLC fingerprints of raw jujube honey samples.

Fig. 3 illustrates the HPLC fingerprint for 15 samples of jujube honey, showing four prominent peaks with similarities between 0.81 and 0.94 (Table S2). Four prominent peaks, specifically 4-hydroxy-8-methoxyquinoline, vomifoliol, indole-3-carboxaldehyde, and jujubin, can be identified as the key fingerprint characteristics (Fig. 3). The standard HPLC fingerprints can serve as nontarget authenticity criteria for Chinese jujube honeys by comparing them with the HPLC profiles of commercial honey samples.

### 3.3 Authenticity assessments of commercial jujube honey samples

The evaluation criteria were established based on the data from raw jujube honey samples to assess honey quality. Primarily, the HPLC profile of commercial samples should resemble the standard HPLC fingerprints in Fig. 3. Furthermore, each characteristic compound needs to satisfy the following criteria: the levels of 4-hydroxy-8-methoxyquinoline, vomifoliol, indole-3-carboxaldehyde, and jujubin should be higher than 0.65, 4.50, 1.34, and 0.15 mg/kg, respectively.

**Fig. 4:**
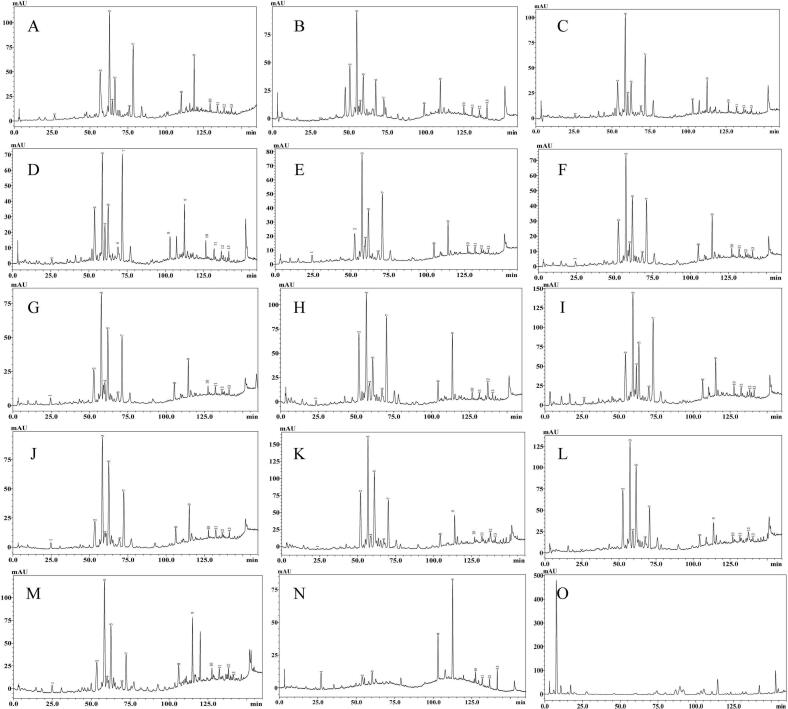
HPLC profiles of commercial jujube honey samples (260 nm).

The HPLC profiles and quantitative results of the commercial honey samples are shown in Fig. 4 and Table 3. These samples can be classified into three groups. Compared with the standard HPLC fingerprint (Fig. 3), the first group exhibited similar HPLC profiles (Fig. 4), including brands A to M. For these 13 samples, the concentrations of 4-hydroxy-8-methoxyquinoline, vomifoliol, indole-3-carboxaldehyde, and jujubin were significantly higher than the minimum level of raw jujube honeys (Table 3), indicating that these 13 commercial samples were genuine jujube honeys. The HPLC profile of brand N was dissimilar to the standard HPLC fingerprint, and only nine valid chromatographic peaks were detected, indicating that Brand N may be impersonated as jujube honey by other honeys. Notably, the HPLC profile of brand O was remarkably dissimilar to the standard HPLC fingerprint, and no valid chromatographic peak was detected. This commercial honey sample appears to be fake jujube honey with syrup.

**Table 3:** Contents of characteristic compounds in commercial honey samples (mg/kg).

Compound	C1	C2	C3	C4	C5	C6	C7	C8
4-Hydroxyquinoline	0.30 ± 0.02	0.21 ± 0.01	0.21 ± 0.01	0.26 ± 0.02	0.23 ± 0.02	0.19 ± 0.01	0.25 ± 0.02	0.29 ± 0.01
4-Hydroxy-8-methoxyquinoline	1.04 ± 0.07	0.99 ± 0.07	1.10 ± 0.07	0.87 ± 0.05	0.67 ± 0.04	0.71 ± 0.05	0.76 ± 0.06	1.21 ± 0.13
Vomifoliol	7.81 ± 0.48	8.04 ± 0.55	6.19 ± 0.54	5.85 ± 0.28	6.13 ± 0.55	5.48 ± 0.51	7.39 ± 0.56	9.31 ± 0.82
2-Hydroxyquinoline	0.98 ± 0.06	0.62 ± 0.04	0.78 ± 0.05	0.54 ± 0.04	0.43 ± 0.02	0.39 ± 0.04	0.51 ± 0.05	1.07 ± 0.09
Indole-3-carboxaldehyde	2.23 ± 0.13	2.15 ± 0.15	1.98 ± 0.15	1.91 ± 0.15	2.02 ± 0.17	2.14 ± 0.15	1.85 ± 0.14	2.04 ± 0.21
Jujubin	0.46 ± 0.02	0.51 ± 0.04	0.35 ± 0.02	0.24 ± 0.01	0.18 ± 0.01	0.23 ± 0.03	0.18 ± 0.01	0.72 ± 0.05
Phaseic acid	2.02 ± 0.14	0.64 ± 0.04	1.76 ± 0.13	0.34 ± 0.02	0.92 ± 0.06	0.86 ± 0.07	0.93 ± 0.06	1.79 ± 0.15
*Trans, trans*-abscisic acid	0.36 ± 0.02	0.39 ± 0.02	0.54 ± 0.03	0.70 ± 0.05	0.57 ± 0.04	0.49 ± 0.03	0.53 ± 0.04	0.48 ± 0.04
*Cis, trans*-abscisic acid	0.85 ± 0.04	1.12 ± 0.08	0.87 ± 0.05	0.33 ± 0.02	0.71 ± 0.08	0.69 ± 0.05	0.87 ± 0.06	0.93 ± 0.07
N1(Z), N5(Z), N10(Z)-tri*-p*-coumaroyl spermidine	0.42 ± 0.03	0.69 ± 0.03	0.73 ± 0.05	0.62 ± 0.04	0.58 ± 0.04	0.68 ± 0.05	0.43 ± 0.04	0.51 ± 0.05
N1(Z), N5(Z), N10(E)-tri-*p*-coumaroyl spermidine	0.67 ± 0.03	0.45 ± 0.02	0.86 ± 0.05	0.92 ± 0.07	0.47 ± 0.03	0.81 ± 0.05	0.58 ± 0.06	0.74 ± 0.06
N1(E), N5(Z), N10(E)-tri-*p*-coumaroyl spermidine	0.62 ± 0.03	0.71 ± 0.05	0.75 ± 0.04	1.23 ± 0.13	0.86 ± 0.08	0.95 ± 0.07	0.63 ± 0.03	0.71 ± 0.07
N1(E), N5(E), N10(E)-tri-*p*-coumaroyl spermidine	0.50 ± 0.03	1.05 ± 0.05	0.63 ± 0.04	0.94 ± 0.08	0.91 ± 0.08	0.78 ± 0.06	0.95 ± 0.04	0.58 ± 0.04

Compound	C9	C10	C11	C12	C13	C14	C15	
4-Hydroxyquinoline	0.28 ± 0.01	0.24 ± 0.02	0.33 ± 0.02	0.25 ± 0.02	0.23 ± 0.01	1.25 ± 0.08	–	
4-Hydroxy-8-methoxyquinoline	1.33 ± 0.11	0.71 ± 0.05	1.37 ± 0.11	1.18 ± 0.09	0.93 ± 0.06	0.42 ± 0.03	–	
Vomifoliol	10.95 ± 0.81	8.83 ± 0.62	11.58 ± 0.94	9.57 ± 0.72	8.47 ± 0.79	–	–	
2-Hydroxyquinoline	1.19 ± 0.10	0.81 ± 0.06	1.13 ± 0.07	1.07 ± 0.09	0.82 ± 0.06	–	–	
Indole-3-carboxaldehyde	2.13 ± 0.19	1.95 ± 0.16	1.85 ± 0. 16	1.83 ± 0.15	1.91 ± 0.18	0.68 ± 0.03	–	
Jujubein	0.97 ± 0.06	0.27 ± 0.02	0.91 ± 0.07	0.82 ± 0.06	0.56 ± 0.04	–	–	
Phaseic acid	1.62 ± 0.13	0.89 ± 0.07	1.83 ± 0.16	1.53 ± 0.12	1.39 ± 0.12	–	–	
*Trans, trans*-abscisic acid	0.59 ± 0.05	0.45 ± 0.03	0.62 ± 0.05	0.37 ± 0.02	0.45 ± 0.04	0.74 ± 0.03	–	
*Cis, trans*-abscisic acid	0.81 ± 0.07	0.98 ± 0.08	0.97 ± 0.06	0.58 ± 0.06	0.76 ± 0.06	2.26 ± 0.13	–	
N1(Z), N5(Z), N10(Z)-tri-*p*-coumaroyl spermidine	0.61 ± 0.04	0.51 ± 0.03	0.46 ± 0.03	0.67 ± 0.04	0.52 ± 0.04	0.51 ± 0.03	–	
N1(Z), N5(Z), N10(E)-tri-*p*-coumaroyl spermidine	1.18 ± 0.09	0.39 ± 0.02	1.05 ± 0.08	0.81 ± 0.05	0.67 ± 0.05	0.84 ± 0.06	–	
N1(E), N5(Z), N10(E)-tri-*p*-coumaroyl spermidine	0.79 ± 0.06	0.82 ± 0.05	0.82 ± 0.07	0.76 ± 0.06	0.84 ± 0.06	0.75 ± 0.05	–	
N1(E), N5(E), N10(E)-tri-*p*-coumaroyl spermidine	0.63 ± 0.04	0.93 ± 0.05	0.75 ± 0.06	0.63 ± 0.05	0.69 ± 0.05	0.69 ± 0.04	–	

Note: “-” represents “not detected”.

## 4 Discussion

Honey is frequently adulterated and consistently ranks among the top ten most reported food fraud items in the EU, alongside milk, olive oil, tea, and coffee (Schwolow, Gerhardt, Rohn, & Weller, 2019). This fraudulent activity harms consumer interests and undermines beekeepers' motivation, contributing to the decline in honeybee populations and damaging the ecosystem. Therefore, it is crucial to implement strategies to identify and prevent honey fraud. Traditional adulteration techniques, such as syrup blending, complicate the detection of all types of adulteration when relying solely on syrup markers. Consequently, a novel and reliable analytical method needs to be developed to identify and quantify characteristic components. This will help establish accurate assessment criteria and prevent misjudgment.

The secondary metabolites vary significantly among plants; some unique components are considered characteristics. A reliable strategy is to identify some intrinsic and specific components that reflect the plant source of honey. Another method is to use nontargeted HPLC fingerprints to detect every component. This study utilized targeted characteristic components and nontargeted HPLC fingerprints to verify honey authenticity. We identified 4-hydroxy-8-methoxyquinoline, vomifoliol, indole-3-carboxaldehyde, and jujubin as the targeted characteristic compounds. Subsequently, we analyzed the chromatographic characteristics of phytochemicals in all raw honey samples and generated the nontargeted HPLC fingerprints. We established evaluation criteria (Result 3.3) to assess the authenticity of commercial honey by analyzing characteristic components and comparing HPLC fingerprints. Our results indicate that most of the fifteen commercial jujube honey samples were genuine honey that complied with the evaluation criteria, except samples N and O. Moreover, we speculated that the adulteration in brand N was due to the misidentification of nectar plants, while the adulteration in brand O involved pure syrup adulteration. In our previous study, this evaluation criteria based on characteristic component analysis and HPLC fingerprint were able to significantly identify the authenticity of a variety of honeys, including rape, acacia, linden, safflower, Chinese sumac, bauhinia longan, litchi, and schefflera honeys (Du et al., 2023; Qiao et al., 2020; Zhu et al., 2024). This study further confirmed the reliability of this evaluation criteria and provided supporting evidence for its necessity in identifying unknown compounds in honey authenticity verification.

We identified 13 major phytochemicals in Chinese *Ziziphus jujuba* honey. Some phytochemicals are potentially present in most honeys, including two abscisic acids and four tri-*p*-coumaroyl spermidines. Abscisic acid, an important plant hormone, is widely present in plant flowers and crucial in regulating the growth of flowers and fruits (K. Kim et al., 2022). As a result, it is easily carried into honey by bees and has been detected in various types of honey (Elisabetta Schievano, Morelato, Facchin, & Mammi, 2013). Additionally, the tri-*p*-coumaroyl spermidines mainly originated from the pollen in the honey (Qiao et al., 2023), and more and more studies have shown that honeys contain tri-*p*-coumaroyl spermidines (Liu, Qiao, Ning, Huang, & Luo, 2023). Therefore, these phytochemicals are not suitable as the intrinsic markers of monofloral honeys. Other phytochemicals have been reported in some types of honey, such as 4-hydroxyquinoline, vomifoliol, 2-hydroxyquinoline, and phaseic acid. 4-Hydroxyquinoline was reported in the cornflower and *castanopsis* honey, with the average contents being 2.1 and 2.03 mg/kg (Oelschlaegel et al., 2012; Yu et al., 2023), higher than that of jujube honey. 2-Hydroxyquinoline was identified in 8 types of Ethiopian honey (Morlock, Belay, Heil, Mehl, & Borck, 2022); and phaseic acid was reported in snowbell, acacia and Chinese sumac honey (J.-G. Kim et al., 2021). The levels of these phytochemicals in jujube honey were not significantly different from those in other types of honey. Consequently, 4-hydroxyquinoline, 2-hydroxyquinoline, and phaseic acid were not suitable for being considered as characteristic phytochemicals of jujube honey. Noteworthily, vomifoliol was quantified in Blue Gum (*Eucalyptus leucoxylon*) and Yellow Box (*Eucalyptus melliodora*) honeys, with an average content of 0.3 mg/kg in these honeys (D'Arcy, Rintoul, Rowland, & Blackman, 1997), which is significantly lower than the average content of 10.25 mg/kg in jujube honey. Therefore, although vomifoliol has been reported in eucalyptus honeys, it can still be considered as the characteristic phytochemical of jujube honey due to its higher content. To our knowledge, some distinguished phytochemicals, including 4-hydroxy-8-methoxyquinoline, indole-3-carboxaldehyde, and jujubin, were first identified in honey. Thus, 4-hydroxy-8-methoxyquinoline, vomifoliol, indole-3-carboxaldehyde, and jujubin can be considered as the characteristic phytochemical of jujube honey. In previous studies, researchers identified 15 phenolic substances in jujube honey, including protocatechuic acid, chlorogenic acid and caffeic acid (Yang, Zhao, Xu, Yang et al., 2021). The component with the highest content was protocatechuic acid, with a content of 0.11–0.16 mg/kg, which was significantly lower than the content of any component identified in this study, indicating that these 15 phenolic substances may not be the main phytochemicals in jujube honey. In Moroccan jujube honeys, researchers identified six components: 4-hydroxyquinoline glucoside, 4-hydroxyquinoline, kynurenic acid, *p*-hydroxybenzoic acid, caffeic acid and methyl syringate (Khallouki, Akdad, Bouddine, Hajji, & Owen, 2020). However, most of these components have been reported in other honeys, such as 4-hydroxyquinoline in cornflower and *castanopsis* honey (Oelschlaegel et al., 2012; Yu et al., 2023), kynurenic acid in chestnut honey(Turski et al., 2016), caffeic acid in Tualang honey (Khalil, Alam, Moniruzzaman, Sulaiman, & Gan, 2011), and *p*-hydroxybenzoic acid and methyl syringate in rapeseed honey (Qiao et al., 2020), and only 4-hydroxyquinoline glucoside would be a characteristic component of jujube honey. Consequently, this study not only expands the characteristic phytochemical profile of jujube honey but also establishes novel authentication criteria for its botanical authenticity, thereby significantly advancing research on honey origin verification through these newly identified molecular markers.

Recently, many studies have focused on identifying most phytochemicals in honey using nontargeted metabolomics approaches, such as GC–MS/MS and HPLC-QTOF-MS/MS. The basic process involves dissolving a small amount of the sample, analyzing it using GC–MS/MS and HPLC-QTOF-MS/MS to obtain data, comparing the results with a database, and applying algorithms to identify components with varying concentrations in the honey (Zhao et al., 2023). Nontargeted metabolomics can detect a wide range of components in honey at high throughput, including sugars, phenols, terpenes, and amino acids (N. Li et al., 2024). While this metabolomics method, combined with databases and algorithms, can partially differentiate the components of specific monofloral honey from those of other monofloral honeys, it still has certain limitations. On the one hand, since both GC–MS/MS and HPLC-QTOF-MS/MS employ electron impact mass spectrometry (EI-MS) or electrospray ionization mass spectrometry (ESI-MS), they generate numerous fragment ions. The ion fragments and cleavage patterns of the same compound can vary depending on the ionization voltage and the specific instrument used. However, some data processing software programs may misidentify these fragment ions as the molecular ions of honey compounds, resulting in a significant amount of false positive data, and the same algorithm can yield different results under these conditions. On the other hand, nontargeted metabolomics based on data processing software cannot identify isomers. Since isomers exhibit almost identical ion fragments and fragmentation patterns, data processing software may incorrectly identify them as the same substance; this problem can also arise from incomplete separation during HPLC. In this study, three groups of isomers were identified, including 4-hydroxyquinoline and 2-hydroxyquinoline, *trans, trans*-abscisic acid and *cis, trans*-abscisic acid, and four tri-*p*-coumaroyl spermidines, based on complete HPLC separation and distinct maximum UV absorption wavelengths. However, the data processing software can only identify them as the 4-hydroxyquinoline, abscisic acid and tri-coumaroyl spermidine (Yu et al., 2023). Most importantly, although nontargeted metabolomics provides a lot of data on phytochemicals, only a fraction of it is represented in existing databases. Consequently, metabolomics cannot identify compounds that are absent from these databases. In other words, nontargeted metabolomics cannot discover the new component. In this study, jujubin was identified in jujube honey and it had only been reported once before in Japanese tobacco (Takagi, Fujimori, Kaneko, & Kato, 1981). The MSMS data of jujubin is not listed in any database, making it impossible to identify using untargeted metabolomics Thus, a more reliable approach to identifying phytochemicals should involves the following steps: complete separation of components using HPLC, identification of each peak's components via MSMS data, and verification with commercial standards. The indispensable step in the MSMS data identification process is determining the molecular weight of the compound through the quasi-molecular ion peak (Hissong, Evans, & Evans, 2023). Thus, we suggest the combination of nontargeted metabolomics with targeted HPLC-PAD-MSMS to identify the characteristic components.

## 5 Conclusion

In this research, 13 phytochemicals were identified and quantified in *Ziziphus jujuba* honey, including 5 terpenoids, 4 phenolamides, 3 quinolines and 1 indole. To the best of our knowledge, 4-hydroxy-8-methoxyquinoline, indole-3-carboxaldehyde, and jujubin were new findings in honey. Vomifoliol represents the most abundant in jujube honey samples, with a concentration significantly higher than that in other monofloral honeys. Therefore, 4-hydroxy-8-methoxyquinoline, indole-3-carboxaldehyde, jujubin, and vomifoliol are considered as the characteristic components of jujube honey. In addition, we establish evaluation criteria for jujube honey based on the contents of characteristic components and HPLC fingerprints. Based on the standard, the adulterated samples are distinguished from genuine jujube honey. This approach appears to offer substantial improvements over conventional detection methods for the authentication of *Ziziphus jujuba* honey.

## CRediT authorship contribution statement

**Hequan Zhu:** Writing – original draft, Methodology, Formal analysis. **Liqiang Liu:** Visualization, Investigation, Data curation. **Rongshen Wang:** Investigation, Conceptualization. **Zijing Wang:** Investigation, Conceptualization. **Yuesen Wang:** Investigation, Conceptualization. **Jie Dong:** Visualization, Investigation, Data curation. **Qiqi Wang:** Visualization, Investigation, Data curation. **Jiangtao Qiao:** Writing – review & editing, Validation, Resources, Methodology, Funding acquisition, Data curation, Conceptualization. **Hongcheng Zhang:** Writing – review & editing, Resources, Funding acquisition, Conceptualization.

## Declaration of competing interest

The authors declare that they have no known competing financial interests or personal relationships that could have appeared to influence the work reported in this paper.

## Data Availability

Data will be made available on request.

## Appendix A Supplementary data

Supplementary material Image 1
