# Supplementary material for: Unveiling the phytochemical profiles of Ziziphus jujuba honey: An authenticity assurance approach
Source: Food Chem X. 2025 Jun 3;29:102622. doi: 10.1016/j.fochx.2025.102622 (PMC12182770; doi:10.1016/j.fochx.2025.102622)
Supplement: Supplementary file 1 — Supplementary material [file mmc1.pdf]

Unveiling the Phytochemical Profiles of *Ziziphus jujuba* Honey: An Authenticity Assurance Approach

Hequan Zhu[a,b,f], Liqiang Liu[a], Rongshen Wang[c], Zijing Wang[d],Yuesen Wang[e], Jie Dong[b,f], Qiqi Wang[b,f], Jiangtao Qiao[a, *], Hongcheng Zhang[b,f,*]

[a] School of Life Sciences and Food Engineering, Hebei University of Engineering, Handan 056038, China;

[b] State Key Laboratory of Resource Insects, Institute of Apicultural Research, Chinese Academy of Agricultural Sciences, Beijing 100093, China;

[c] Shijiazhuang Center for Animal Disease Prevention and Control, Shijiazhuang, 130100, China;

[d] College of Bee Science and Biomedicine, Fujian Agriculture and Forestry University, Fuzhou, 350002, China.

[e] Hebei Ruiyuan Beekeeping Co., Ltd., Shijiazhuang, 051230, China;

[f] Key Laboratory of Bee Products for Quality and Safety Control, Ministry of Agriculture and Rural Affairs, Beijing 100093, China;

*Corresponding author:

Email: 506996362@qq.com (JQ)

460414874@qq.com (HZ)

[*]These authors also contributed equally to this work.

Supplementary Table 1. Characterization of the analyzed raw honey samples

Sample	Type of honey	Botanical origin	Predominant pollen (%)	Geographical origin	Production year
Sample1	Jujube honey	*Ziziphus jujuba* Mill.	75	Lvliang City, Shanxi	2022
Sample2	Jujube honey	*Ziziphus jujuba* Mill.	80	Yuncheng City, Shanxi	2022
Sample3	Jujube honey	*Ziziphus jujuba* Mill.	64	Jinzhong City, Shanxi	2022
Sample4	Jujube honey	*Ziziphus jujuba* Mill.	67	Yulin City, Shaanxi	2022
Sample5	Jujube honey	*Ziziphus jujuba* Mill.	72	Weinan City, Shaanxi	2022
Sample6	Jujube honey	*Ziziphus jujuba* Mill.	70	Xianyang City, Shaanxi	2022
Sample7	Jujube honey	*Ziziphus jujuba* Mill.	65	Zhengzhou City, Henan	2022
Sample8	Jujube honey	*Ziziphus jujuba* Mill.	78	Sanmenxia City, Henan	2022
Sample9	Jujube honey	*Ziziphus jujuba* Mill.	77	Anyang City, Henan	2022
Sample10	Jujube honey	*Ziziphus jujuba* Mill.	74	Shijiazhuang City, Hebei	2022
Sample11	Jujube honey	*Ziziphus jujuba* Mill.	63	Cangzhou City, Hebei	2022
Sample12	Jujube honey	*Ziziphus jujuba* Mill.	68	Baoding City, Hebei	2022
Sample13	Jujube honey	*Ziziphus jujuba* Mill.	76	Hami City, Xinjiang	2022
Sample14	Jujube honey	*Ziziphus jujuba* Mill.	69	Hotan Prefecture, Xinjiang	2022
Sample15	Jujube honey	*Ziziphus jujuba* Mill.	73	Aksu Prefecture, Xinjiang	2022

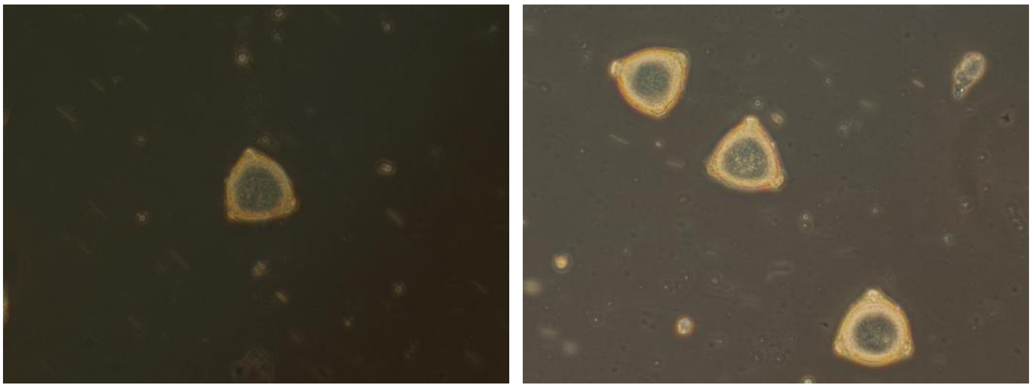

Supplementary Figure 1. Pollen grain photomicrographs of monofloral jujube honeys

Supplementary Table 2. Similarity analysis among Jujube raw honey samples

	S1	S2	S3	S4	S5	S6	S7	S8	S9	S10	S11	S12	S13	S14	S15
S1	1	0.89	0.91	0.89	0.81	0.85	0.84	0.91	0.83	0.94	0.93	0.81	0.89	0.92	0.94
S2	0.89	1	0.83	0.82	0.86	0.83	0.84	0.81	0.92	0.89	0.93	0.83	0.82	0.91	0.85
S3	0.91	0.83	1	0.94	0.85	0.84	0.82	0.81	0.87	0.81	0.91	0.81	0.87	0.84	0.82
S4	0.89	0.82	0.94	1	0.81	0.89	0.83	0.86	0.81	0.89	0.88	0.92	0.91	0.83	0.86
S5	0.81	0.86	0.85	0.81	1	0.83	0.89	0.81	0.91	0.81	0.87	0.85	0.81	0.93	0.89
S6	0.85	0.83	0.84	0.89	0.83	1	0.83	0.89	0.83	0.85	0.86	0.87	0.89	0.82	0.83
S7	0.84	0.84	0.82	0.83	0.89	0.83	1	0.83	0.82	0.84	0.82	0.86	0.83	0.89	0.93
S8	0.91	0.81	0.81	0.86	0.81	0.89	0.83	1	0.86	0.82	0.89	0.81	0.86	0.84	0.82
S9	0.83	0.92	0.87	0.81	0.91	0.83	0.82	0.86	1	0.85	0.87	0.81	0.86	0.84	0.82
S10	0.94	0.89	0.81	0.89	0.81	0.85	0.84	0.82	0.85	1	0.87	0.81	0.89	0.82	0.82
S11	0.93	0.93	0.91	0.88	0.87	0.86	0.82	0.89	0.87	0.87	1	0.87	0.81	0.93	0.82
S12	0.81	0.83	0.81	0.92	0.85	0.87	0.86	0.81	0.81	0.81	0.87	1	0.84	0.91	0.92
S13	0.89	0.82	0.87	0.91	0.81	0.89	0.83	0.86	0.86	0.89	0.81	0.84	1	0.93	0.83
S14	0.92	0.91	0.84	0.83	0.93	0.82	0.89	0.84	0.84	0.82	0.93	0.91	0.93	1	0.89
S15	0.94	0.85	0.82	0.86	0.89	0.83	0.93	0.82	0.82	0.82	0.82	0.92	0.83	0.89	1
